# Selective Neural Electrical Stimulation of an Injured Facial Nerve Using Chronically Implanted Dual Cuff Electrodes

**DOI:** 10.3390/brainsci12111457

**Published:** 2022-10-27

**Authors:** Arash Abiri, Steven Chau, Nathan R. James, Khodayar Goshtasbi, Jack L. Birkenbeuel, Ronald Sahyouni, Robert Edwards, Hamid R. Djalilian, Harrison W. Lin

**Affiliations:** 1 Department of Otolaryngology–Head and Neck Surgery, University of California, Irvine Medical Center, Orange, CA 92868, USA; 2 Department of Pathology and Laboratory Medicine, University of California, Irvine School of Medicine, Irvine, CA 92617, USA

**Keywords:** neuroprosthetics, implantable neural interfaces, flexible neural electrodes, facial nerve, facial paralysis, neurorehabilitation

## Abstract

Facial nerve (FN) injury can lead to debilitating and permanent facial paresis/paralysis (FP), where facial muscles progressively lose tone, atrophy, and ultimately reduce to scar tissue. Despite considerable efforts in the recent decades, therapies for FP still possess high failure rates and provide inadequate recovery of muscle function. In this pilot study, we used a feline model to demonstrate the potential for chronically implanted multichannel dual-cuff electrodes (MCE) to selectively stimulate injured facial nerves at low current intensities to avoid stimulus-induced neural injury. Selective facial muscle activation was achieved over six months after FN injury and MCE implantation in two domestic shorthaired cats (*Felis catus*). Through utilization of bipolar stimulation, specific muscles were activated at significantly lower electrical currents than was achievable with single channel stimulation. Moreover, interval increases in subthreshold current intensities using bipolar stimulation enabled a graded EMG voltage response while maintaining muscle selectivity. Histological examination of neural tissue at implant sites showed no appreciable signs of stimulation-induced nerve injury. Thus, by selectively activating facial musculature six months following initial FN injury and MCE implantation, we demonstrated the potential for our neural stimulator system to be safely and effectively applied to the chronic setting, with implications for FP treatment.

## 1. Introduction

Facial paralysis and paresis (FP) is a devastating condition that impacts roughly 50 per 100,000 people in the United States every year [1]. This condition is most commonly caused by damage to the facial nerve (FN) secondary to surgery, neoplasm, trauma, or infection [2,3]. Those inflicted with FP often present with limited control over the facial muscles that are responsible for their facial expression, communication, oral competency, and blink function [3]. Consequently, FN damage can progressively result in hypotonia, hypotrophy, and ultimately, fibrosis of facial musculature. While a majority of patients with FP recover some muscle function, nearly 30% are left with permanent, debilitating dysfunctions such as facial spasms, synkinesis, and asymmetry [4].

As inflammation is an accepted cause of nerve damage, corticosteroid therapy is one of the most common treatments for acute idiopathic FP [2]. However, this treatment does not address those suffering from permanent FN deficits, whose therapeutic options are limited to surgical intervention and physical therapy. Two common surgical approaches employ prosthetics or autologous muscle grafts to restore some of the diminished muscle function. Despite their satisfactory results, these procedures have a 10–15% failure rate and are limited in their capacity to address deficits beyond precise areas of the face [5,6]. The advent of electrical neural stimulation provides a promising approach for treating both acute and permanent nerve injuries. Through selective stimulation of nerve fascicles, electrical neural stimulation can promote correction of muscle tone and enhance recovery times in FP patients [7,8,9]. Thus, electrical stimulation shows potential in becoming a compelling alternative to current prosthetic and muscle graft-based therapeutic approaches.

Previous studies have investigated implanting neural electrodes to achieve selective muscle recruitment (i.e., activation of a subset of muscles innervated by the same nerve) [10,11]. In recent years, advancements in intraneural electrodes have even enabled selective activation at the level of single fascicles [12]. Extraneural cuff electrodes serve as a less invasive, albeit less precise, alternative to intraneural electrodes by wrapping around the nerve and providing multiple points of contact for stimulation. These cuff electrodes have been applied to selective vagus nerve stimulation in rodent models and demonstrated success in controlling blood pressure, heart rate, and modulating the inflammatory pathway [13,14,15]. The potential for cuff electrodes to achieve selective muscle activation has also been extensively examined in the context of the femoral and sciatic nerves; however, this technology’s application for the facial nerve has not yet been well defined [16,17,18,19]. Recently, our group demonstrated the efficacy of positioning a single multichannel cuff electrode (MCE) at the main trunk of the FN to selectively stimulate fiber populations and predictably activate specific facial muscles [20].

In this study evaluating chronically implanted cuffs, we sought to increase the resolution of this neuro-prosthetic interface by utilizing two concentric 8-channel MCEs at the dorsal (upper branch) and ventral (lower branch) rami of the FN. This was done in the context of permanent unilateral FN paralysis by purposefully producing crush injuries distal to the cuffs implanted in a feline model. We performed epineural stimulation testing six months following a post-implantation recovery period to elucidate the MCEs’ long-term functional capacity and the histological changes associated with their chronic implantation post-FN injury.

## 2. Materials and Methods

### 2.1. Animals

All experiments were conducted in strict accordance with a protocol approved by University of California, Irvine Institutional Animal Care and Use Committee (Protocol Number: AUP-17-189), and in agreement with the National Institutes of Health Animal Welfare Guidelines. A feline model was used for this study due to the similarity in its facial neuromuscular system to that of humans [21]. Moreover, cats possess a highly accessible facial nerve, which provides easier access for stimulation at specific sites along the course of the nerve. Additionally, by virtue of being a larger animal, they have larger and more defined facial musculature that can be more easily targeted for needle electromyographic recordings.

We obtained two female domestic shorthaired cats (*Felis catus*) from a research breeding colony at University of California, Davis. Both cats underwent survival surgeries, in which nerve injuries were induced and electrode implantations were performed. In the first cat (Cat A), electrodes were implanted and stimulated at the lower and upper branches of the left FN. In the second cat (Cat B), to assess the histologic impact of chronic stimulation on an injured nerve, electrodes were implanted at the main trunks of bilateral FNs, but only the electrode implanted on the left FN was chronically stimulated. Postoperatively, the cats were closely monitored for proper breathing, feeding, and hydration as well as for any signs of infection. Routine daily checks were also conducted, where the behaviors of the cats, including any signs of lethargy, loss of appetite, wound guarding, or agitation, were observed in a group housing setting. The animals were fed and weighed daily, and their surgical and implant sites were inspected and cleaned as needed.

### 2.2. Anesthesia

During stimulation experiments, a light anesthetized state was induced by injecting ketamine (20 mg/kg) and acepromazine (1 mg/kg) intramuscularly. Additional doses of ketamine (10 mg/kg) were administered as needed to maintain stable sedation.

### 2.3. MCE Specifications

Four custom 8-channel MCEs were provided by MicroProbes for Life Science (Gaithersburg, MD, USA). Each cuff had an inner diameter of 1.5 mm, with two separate rings of four, 100 × 100 µm, rectangular (tripolar) platinum contacts arranged concentrically every 90 degrees (0°, 90°, 180°, 270°) within a silicon enclosure. The arrangement of the rings and contacts allowed for monopolar stimulation of unique spatial locations on the nerve. The second parallel ring enabled bipolar stimulation, in which two electrodes of the same cuff could be stimulated simultaneously to elicit an amplified response. Current intensity at the maximum charge injection capacity (CIC) was calculated to be 1.22 mA using the average reported value of CIC for platinum electrodes using cathodic first pulses (100–150 µC/cm^2^) [22] applied to the implanted contact surface area (0.0001 cm^2^) for pulse widths of 82 µs. The electrode impedance was measured to be 0.5 kΩ at 1000 Hz using the internal impedance checking function of equipment from Tucker-Davis Technologies System 3 (Alachua, FL, USA).

### 2.4. Surgery: Nerve Injury and MCE Implantation

**Figure 1:**
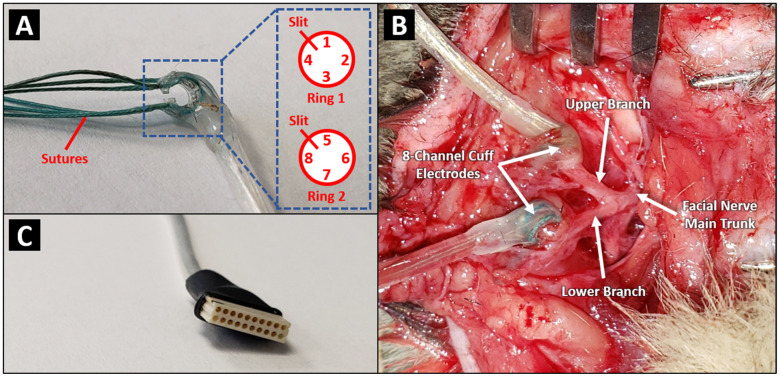
(**A**) 8-channel MCE consisting of 2 parallel electrode rings, in each of which 4 rectangular platinum electrodes were arranged 90 degrees apart within a silicon enclosure. (**B**) Intra-operative image of the two MCEs implanted at the upper and lower FN branches of Cat A. (**C**) Photograph of the male Omnetics connector originating from the MCE in (A).

Survival surgeries were performed on each cat, during which facial nerve injuries were induced and MCEs (Figure 1A) and Omnetics connectors were implanted. To induce a standardized FN injury, a pre-auricular incision was made, and the FN main trunk along with its upper and lower branches were identified and skeletonized. A single 30-s one-click crush injury was induced at each nerve site using a serrated hemostat. In Cat A, sites of crush injuries were at the upper and lower branches of the left FN. In Cat B, crush injuries were induced at the main trunks of both the left and right FNs. MCEs were implanted proximal to the sites of injury (Figure 1B). In Cat A, MCE 1 was implanted at the lower division and MCE 2 was implanted at the upper division. An Omnetics connector encased within a stainless-steel cylinder was also fitted and sealed to the skull using acrylic. To ensure that the MCEs had proper contact with the nerve, each of the contact points on the MCEs was stimulated, one at a time, and EMG voltage responses from the four selected facial muscles were assessed by a nerve integrity monitoring system (NIM Response 2.0; Medtronic Inc., Minneapolis, MN, USA). The wire connectors (Figure 1C) from the MCEs were then tunneled through to the skull mounted Omnetics connector (Minneapolis, MN, USA). A plastic cap was placed over the surface of the Omnetics connector to protect connections from any dirt, debris, or fluid. Six months post-implantation, stimulation experiments were carried out using Cat A every 2 weeks for a period of 6 weeks (3 sessions). Biweekly stimulation experiments were performed on Cat B three months post-implantation for a period of 8 weeks. Due to a skin abscess near the Omnetics connector, stimulation experiments were discontinued 2 weeks earlier in Cat A, as the connection to the implanted electrodes was compromised due to local treatment and animal picking at the connector site. Both cats were put under a lightly sedated state during stimulation experiments. 

### 2.5. Terminal Experiment

Terminal procedures were performed to collect nerve tissue samples for histological examination. Through a pre-auricular incision, the FN main trunk and its branches were isolated. In Cat A, nerve segments of the FN upper and lower branches at the MCE sites were extracted. In Cat B, nerve segments were collected from the main trunks of bilateral FNs. All specimens were fixed in 10% formalin before being stained using hematoxylin and eosin (H&E) and Trichrome. A lethal dose of barbiturate was used for euthanization.

### 2.6. Stimulus Generation and Electromyography (EMG)

A custom optically isolated 16-channel current source, controlled by a 16-bit digital-to-analog converter (Tucker-Davis Technologies RX8, Alachua, FL, USA), was utilized to transmit electrical pulses to the neural electrodes. The stimulator was AC coupled to ensure charge recovery and its current resolution was 1.5 µA, pulse width resolution was 41 µs/phase, and maximum available compliance voltage was 58 V peak-to-peak. Stimulation parameters were controlled using an in-house MATLAB (Natick, MA, USA) program. Stimuli were charge-balanced, symmetric biphasic electrical pulses, initially cathodic, 41 µs per phase, with no interphase gap. To facilitate EMG measurement, electrical pulses were transmitted to the FN every second rather than a continuous train of pulses. In Cat A, channels 1–8 (MCE 1) and channels 9–16 (MCE 2) were stimulated for 10 seconds with different currents ranging from 15 to 60 µA. In Cat B, 100 µA electrical pulses were transmitted for 5 minutes through the electrode implanted on the left FN main trunk.

EMG measurements of the stimulated hemiface were collected via four monopolar needle EMG electrodes (recording area 0.28 mm^2^) placed in four muscles innervated by the upper or lower branches of the FN: levator auris longus (levator), orbicularis oculi (oculi), nasalis, and platysma (Figure S1). The reference and ground electrodes were placed in the lateral/long triceps head on the front limb of the cat. During stimulation experiments, stimulus artifacts in the EMG recordings were confined to the first 1 ms of data. Ten sweeps per condition were collected. The sampling frequency of the raw EMG signal was 24,414 Hz. The raw data underwent high-pass filtering (cut-off frequency = 10Hz), followed by down-sampling to 3052 Hz. Finally, to further enhance signal to noise ratio, a 4th-order bandpass Butterworth filter (100–1500Hz) was applied to the average of the 10 sweeps. The root mean square (RMS) of the EMG response for each muscle was calculated over the duration of the M-wave (from 2 ms to 80 ms after stimulation).

## 3. Results

**Figure 2:**
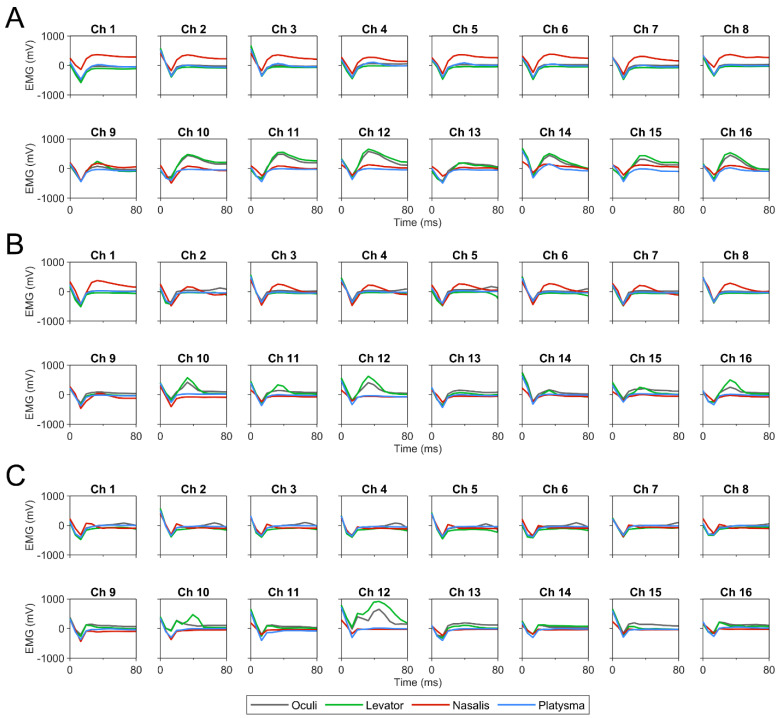
EMG waveforms 6 months post-implantation from four electrically stimulated facial muscles at (**A**) 15, (**B**) 30, and (**C**) 60 µA across 16 channels. Channels 1–8 represented MCE 1 on the lower FN branch. Channels 9–16 represented MCE 2 on the upper FN branch. Stimulation occurred at t = 0 ms.

Electrical stimulation through the two MCEs 6 months post-implantation resulted in selective activation of facial musculature (Figure 2). Levator, nasalis, and oculi were activated when applying a current of 15 µA or higher. However, EMG signals suggested minimal activation of the platysma in either MCE 1 (channels 1–8) or MCE 2 (channels 9–16). While the highest muscle selectivity appeared to occur at 15 µA, the highest stimulation current (60 µA), in contrast, seemed to exhibit a decrease in selective muscle activation, particularly for nasalis in channels 1–8.

**Figure 3:**
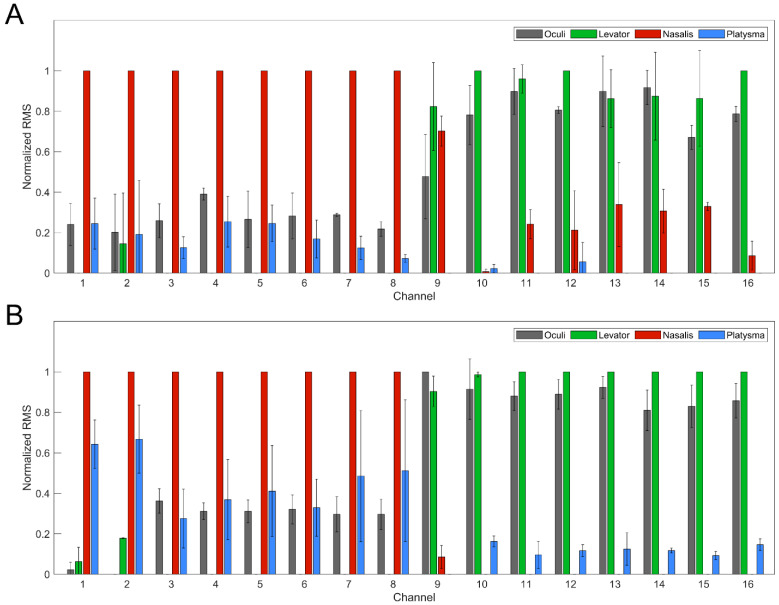
Average normalized RMS from EMG recordings of oculi, levator, nasalis, and platysma for all 16 channels at a current intensity of 15 µA at (**A**) the beginning of stimulation experiments (i.e., 6 months post-implantation) and (**B**) the end of the study. Stimulation of channels 1–8 resulted in activation of nasalis and platysma, while stimulation of channels 9–16 produced selective activation of oculi and levator. Error bars represent 1 standard deviation.

The muscle selectivity of each MCE was evaluated by calculating the normalized RMS of the EMG signals corresponding to stimulation of channels 1–16 at 15 µA. (Figure 3). Channels 1–8 belonging to MCE 1 demonstrated strong selectivity for nasalis and mild-moderate activation of platysma. In contrast, channels 9–16 corresponding to MCE 2 were particularly selective for levator and oculi. Interestingly, at 6 months post-implantation, channel 9 stimulation also exhibited moderate activation of nasalis (Figure 3A). However, this was not apparent in stimulation experiments at the end of the study period (Figure 3B). Although the activation of platysma was relatively low across all 16 channels, the average EMG response was notably higher in channels 1–8 (MCE 1) than channels 9–16 from MCE 2. 

**Figure 4:**
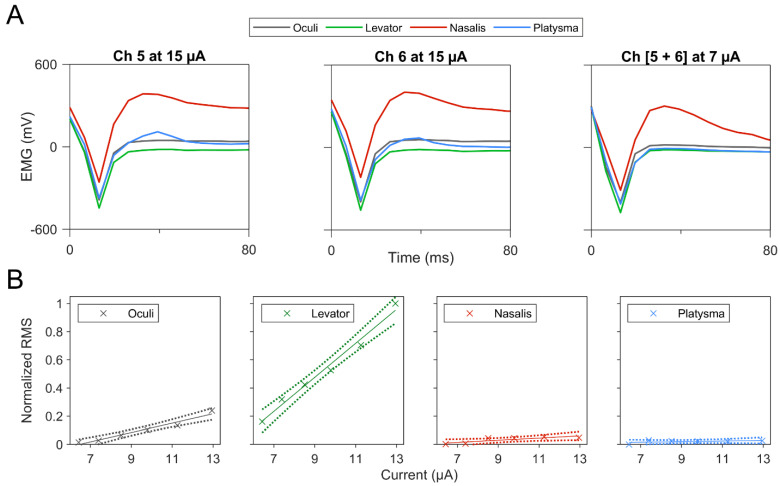
(**A**) Concurrent stimulation of channels 5 and 6 at a reduced current elicited similar activation of nasalis compared to independent stimulation of the channels. (**B**) Graded concurrent stimulation of channels 15 and 16 demonstrated a selective increase in EMG response for oculi and levator. Linear regressions (solid lines) and 95% confidence intervals (dotted lines) were derived from EMG measurements (“×” marks).

To investigate the feasibility of utilizing bipolar stimulation to reduce stimulation current levels while maintaining selective muscle activation, pairs of MCE channels were simultaneously stimulated at significantly reduced (sub-threshold) currents and corresponding EMG signals were analyzed (Figure 4A). Concurrent stimulation of channels 5 and 6 at a current 2.1 times less than that used in individual stimulations yielded similar levels of nasalis activation. Additionally, concurrent stimulation of channels 15 and 16 at 6.4, 7.4, 8.5, 9.8, 11.3, and 12.9 µA demonstrated selective increase in EMG responses for levator and oculi at higher current intensities (Figure 4B). Selective activation of the levator is demonstrated in Video S1, where channels 15 and 16 were concurrently stimulated at approximately 9.8, 11.3, and 12.9 µA.

**Figure 5:**
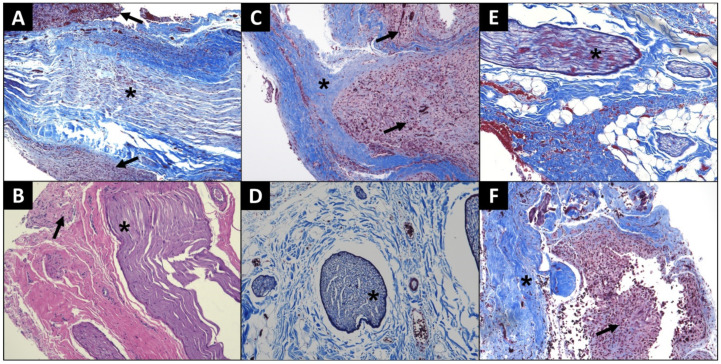
Light microscopy images of facial nerve collected after terminal experiments of Cat A (**A**–**D**) and Cat B (**E**,**F**). (**A**) Left upper facial nerve (star) section collected adjacent to electrode and nerve injury site demonstrating granulomatous tissue formation (arrow) with neutrophilic infiltrate; Trichrome, 10×. (**B**) Left lower facial nerve (star) adjacent to electrode and nerve injury site showed increased collagen between nerves with peripheral neutrophil infiltrate and granulation tissue (arrow); H&E, 10×. (**C**) Left facial nerve (star) adjacent to nerve cuff implant/nerve injury site which received chronic stimulation demonstrating peripheral buildup of granulation tissue (arrow); Trichrome, 10×. (**D**) Right upper facial nerve (star) that received no injury and no electrode implant. This section was taken from approximately the same level as the left sided implant and shows normal tissue morphology; Trichrome, 10×. (**E**) Right lower facial nerve (star) that received no injury and no electrode implant. This section was taken from approximately the same level as left sided implant and shows normal tissue morphology; Trichrome, 10×. (**F**) Axonal fibers (star) of right facial nerve adjacent to nerve cuff implant/nerve injury site which received no chronic stimulation demonstrating peripheral buildup of granulation tissue (arrow); Trichrome, 10×. Scale bar = 200 µm.

Following the terminal experiments, histological examination of extracted facial nerve segments were performed in order to identify any potential foreign body response or electrical injury induced by the implanted electrodes (Figure 5). In Cat A, trichrome staining of the left upper FN adjacent to the site of injury and stimulation demonstrated a buildup of granulomatous tissue with leukocyte infiltration suggestive of prior neuronal injury (Figure 5A). Similarly, H&E staining of the same cat’s left lower FN adjacent to the site of injury and stimulation exhibited increased collagen density between nerves with peripheral accumulation of granulation tissue, indicating a similar pattern of tissue injury (Figure 5B). In contrast, trichrome staining of the uninjured, unstimulated right upper (Figure 5D) and lower FNs (Figure 5E) of the same cat showed normal tissue morphology, suggesting that either crush injury or nerve stimulation may have induced the tissue pattern observed in Figure 5A,B. In Cat B, trichrome staining of the stimulated, injured left FN (Figure 5C) and the unstimulated, injured right FN (Figure 5F) both exhibited a buildup of granulomatous tissue similar to that observed in Cat A specimens. Additionally, we observed similar patterns of atrophic changes and perineural thickening in Cat B’s specimens, suggesting that the appreciable histological signs of injury were secondary to the trauma induced from crush injuries rather than chronic electrical stimulation.

## 4. Discussion

In this pilot study, we utilized a feline model to demonstrate the capability of neural cuff electrodes to induce selective contraction of facial musculature up to six months following severe facial nerve injury and electrode implantation. Thus, our system exhibited several key properties of a facial nerve stimulator that lends itself to being a promising therapy for FP. First, our stimulator was shown to be implantable and demonstrated maintenance of function and safety in the chronic setting. Although our prior work has established the feasibility of a single-cuff system [20], in this study, our findings suggested a favorable efficacy and safety in using a chronically implanted dual-cuff electrode model, providing support for future investigations on this application. Second, we demonstrated that our device could not only stimulate major muscles innervated by the facial nerve, but it could leverage bipolar stimulation to selectively activate individual muscles at low stimulation amplitudes. Moreover, by allowing for a lower electrical current, our dual-cuff system enabled lower doses of electrical stimulation while maintaining contraction amplitude, thereby reducing the potential for current-induced nerve injury and improving overall device safety. Finally, through histological examination of chronically stimulated neural tissue, we demonstrated that our system could safely illicit neural stimulation without inducing new injury to the facial nerve.

Therefore, the presented system for selectively stimulating the FN shows the potential for neuroprosthetic technology, specifically MCEs, to remain functional after an extended period of time post-implantation and stimulate individual facial muscles following severe FN injury. This approach shows promise for possible translation into a novel clinical treatment for facial paresis or paralysis. For example, in the setting of hemifacial paralysis following stroke, wherein the peripheral FN is intact, MCE implantation could facilitate efforts in facial reanimation, such as creating a symmetric smile. Of course, the results of this study are still preclinical and limited to the setting of animal models. Further optimization of parameters and investigation of efficacy and safety in animal models are warranted, followed by a limited clinical trial that may elucidate MCE efficacy in selectively activating human facial muscles.

Our EMG data demonstrated that specific muscle groups could be activated using our dual-cuff electrode system. As hypothesized, the upper cuff, MCE 2, was more selective for activation of levator and oculi, with poor activation of the platysma and nasalis. In contrast, the lower cuff, MCE 1, was more selective for the nasalis and platysma. Interestingly, in the first week of experiments (i.e., 6 months post-implantation), channel 9 from MCE 2 appeared to also exhibit nasalis activation. This behavior, however, was not observed at the end of the study period. It is unclear if this was a result of an aberrant neural pathway or due to improper EMG electrode placement. Additionally, EMG measurements from channels 1–8 of MCE 1 suggested mild oculi activity. Given its proximity to platysma, it is possible that these elevated measurements were secondary to nearby muscle movement. This may be supported by oculi’s relatively higher RMS in our analysis of the last stimulation experiment where platysma activity was observed to be notably higher than in the first experiment. Additionally, muscle activation and selectivity, particularly in MCE 1, appeared relatively low at the highest tested current intensity (60 µA). This may have been due to variations in the study environment, including EMG electrode placement, prior stimulations, and level of animal sedation. Nevertheless, the effective selectivity of the MCE, as demonstrated in this and previous works [20,23,24], is encouraging for future investigations and an eventual human implantation trial.

In this study, it was observed that specific channels could be successfully combined (i.e., bipolar stimulation) to allow for specific muscle activation at low current intensities, suggesting a fascicular organization of the lower and upper FN branches. When channels 5 and 6 were paired, there was more than a two times reduction in the current required for stimulation of nasalis. Moreover, when channels 15 and 16 were paired, we were able to not only exhibit selective levator activation at lower stimulation currents, but also demonstrate the ability to adjust the EMG response, which is a coarse proxy for the degree of muscle recruitment. Since previous work [23] on an injured nerve led to decreased muscle activation than an acutely experimented uninjured nerve, this grading mechanism can be implemented as an artificial amplification system in the future. Frigerio et al. has previously reported that even in the context of severe axonal injury in humans, there remain enough neural connections to invoke muscle activation [25]. With the utilization of this grading phenomenon, future human trials can initiate current delivery with much lower amplitudes for a hypothetically safer experimentation. However, bipolar stimulation was not possible for all the muscles, particularly the platysma. This may represent either a non-topographic organization of the nerve bundle, either naturally or as a result of synkinesis, as the cuff was placed proximal to the nerve injury. 

Importantly, on histological examination, signs of neural injury were not suspected to be due to chronic stimulation or electrode implantation, but secondary to crush-induced trauma. While trauma-induced fibrous tissue formation can result in increased impedance and stimulation thresholds to elicit nerve excitation, we were nonetheless able to selectively stimulate facial muscles at minimal currents. This finding is in-line with a previous report from Lefurge et al., which demonstrated that the inflammatory or desmoplastic response to chronic foreign body implantation and electric stimulation did not hinder the ability to elicit muscle activations [26]. Furthermore, Bowman et al. and Freeberg et al. demonstrated that nerve conduction velocities were similar between acutely and chronically implanted electrodes in rabbit, feline, and human [27,28]. Given that many histologic or physiologic consequences of neuroprosthetic placement occur within the first several months following implantation surgery [29,30,31], our ability to selectively stimulate the FN six months post-implantation demonstrates that the direct electrode-to-nerve interface is sufficient to induce muscle contraction despite trauma-induced fibrotic changes. 

With our findings in mind, there are still several limitations to this study that warrant future investigation. Due to the pilot nature of this study and its small sample size, the strengths of our conclusions were limited, and future studies should implement a larger test cohort to further validate reproducibility and reinforce our results’ external validity. Additionally, modifications are warranted to mitigate issues with synkinesis and retrograde stimulation, which are largely dependent on where the electrodes are placed. By placing them distal to the crush injuries in the future, potential issues with synkinesis may be minimized. Furthermore, this distal placement may decrease the possibility of retrograde stimulation. Therefore, future investigations should also examine the influence of electrode location relative to the site of injury. Due to our wired stimulation system, we were unable to perform more frequent stimulation experiments since additional cat sedation was required, which was not possible without compromising animal safety. In future studies, a wireless implantable stimulator is needed so that the effect of repeated stimulation over time may be studied to better elucidate the stability and safety of our implantable neural electrodes. Finally, our assessment of electrode safety was primarily via histology, and additional evaluation modalities are warranted to ensure tissue integrity and health. Hence, future studies may benefit from implementing interval impedance and stimulus threshold experiments, as they could inform of potential fibrotic changes and provide additional insight on device safety.

## 5. Conclusions

By eliciting selective stimulation of individual facial musculature innervated by upper and lower rami of an injured facial nerve, we have demonstrated the potential for a dual cuff multichannel electrode to remain viable and effective for over six months after implantation. In the feline model, using multiple channels in tandem enabled bipolar stimulation, which required significantly less current to achieve similar responses in muscles, particularly in the levator and nasalis muscles, than individual channel stimulations. While further study is needed, the chronic efficacy of implanted dual cuff electrodes in the context of FP shows promise in being used for targeted therapy in improving muscle function, reducing muscle atrophy, and consequently increasing patient quality of life.

## Data Availability

The datasets used and/or analyzed during the current study are available from the corresponding author (H.W.L) on reasonable request.

## Supplementary Materials

The following supporting information can be downloaded at: https://www.mdpi.com/article/10.3390/brainsci12111457/s1; **Video S1.** Selective activation of *levator* using bipolar stimulation of channels 15 and 16 at three current intensities: 9.8; 11.3, and 12.9 µA. Graded concurrent stimulation of the two channels correlated with increased functional muscle movement. **Figure S1.** Diagram of feline hemiface with approximate locations of *platysma* (blue), *nasalis* (red), *oculi* (gray), and *levator* (green). MCE 1 and 2 (orange box) were implanted at the lower and upper branches of the facial nerve (yellow), respectively.
