# Supplementary figures and images for: Selective Neural Electrical Stimulation of an Injured Facial Nerve Using Chronically Implanted Dual Cuff Electrodes
Source: Brain Sci. 2022 Oct 27;12(11):1457. doi: 10.3390/brainsci12111457 (PMC9688741; doi:10.3390/brainsci12111457)

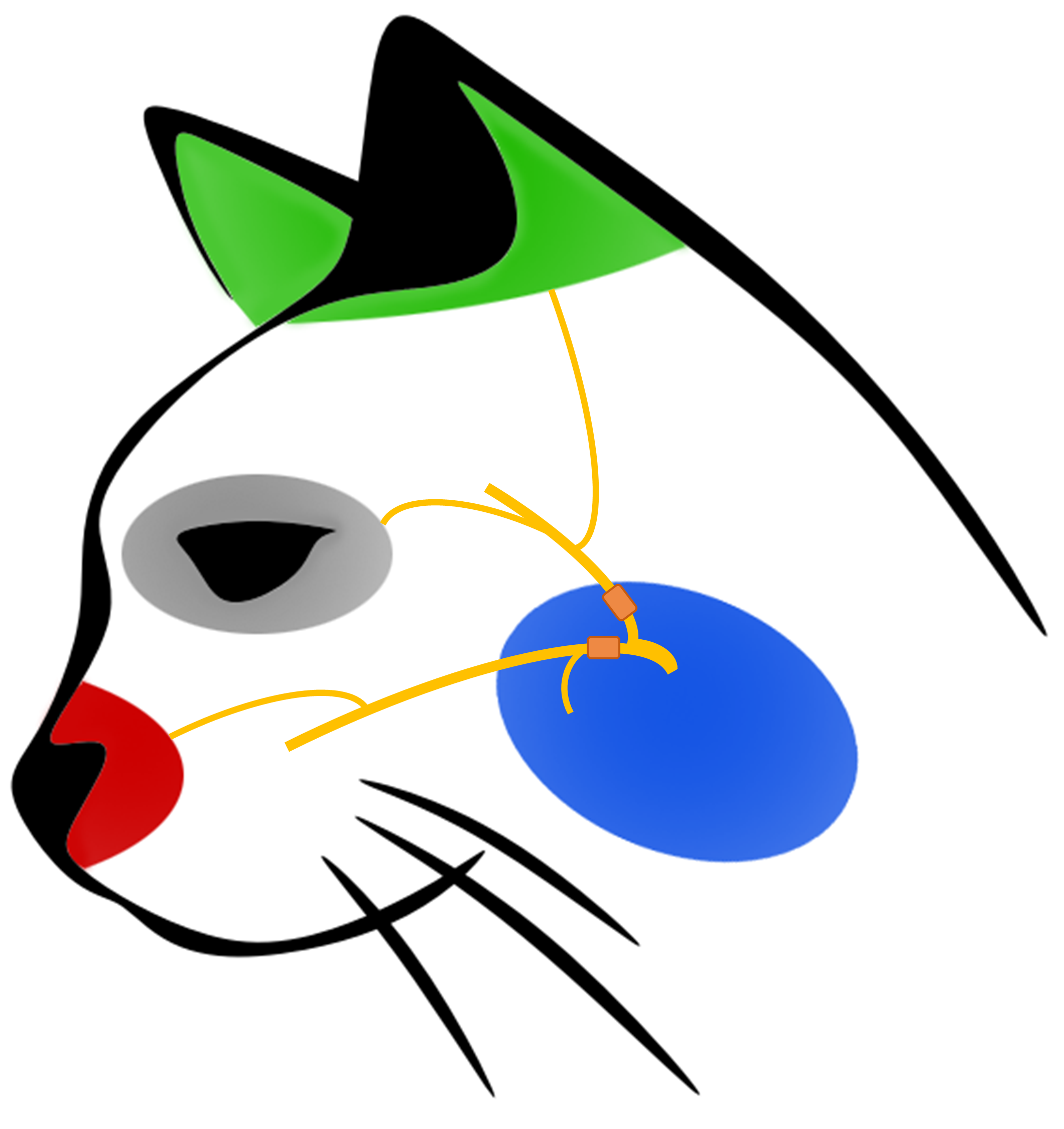

Supplement: Supplementary file 1 [file brainsci-12-01457-s001.zip › Figure S1.tif]
